# Increase in Antibiotic Utilisation in Primary Care Post COVID-19 Pandemic

**DOI:** 10.3390/antibiotics14030309

**Published:** 2025-03-17

**Authors:** Sky Wei Chee Koh, Si Hui Low, Jun Cong Goh, Li Yang Hsu

**Affiliations:** 1 National University Polyclinics, National University Health System, Singapore 119228, Singapore; si_hui_low@nuhs.edu.sg (S.H.L.); jun_cong_goh@nuhs.edu.sg (J.C.G.); 2 Yong Loo Lin School of Medicine, National University of Singapore, Singapore 117597, Singapore; ephhly@nus.edu.sg; 3 Saw Swee Hock School of Public Health, National University of Singapore, Singapore 117549, Singapore

**Keywords:** anti-bacterial agents, antibiotic stewardship, drug prescriptions, primary healthcare, general practice

## Abstract

**Introduction**: The COVID-19 pandemic has disrupted antibiotic use; easing public health measures may alter infection presentations and antibiotic prescribing in primary care. The study investigated post-pandemic antibiotic utilisation trends in primary care. **Methods**: A multi-centre, retrospective cohort study was conducted across seven public primary care clinics in Western Singapore, which included all patients prescribed oral antibiotics between 2022 and 2023. Descriptive statistics were used to visualise the prevalence and conditions of the prescribed antibiotics. Antibiotic quality was evaluated using the WHO’s AWaRe (access, watch, reserve) classification. Antibiotic use was quantified using the number of items dispensed per 1000 inhabitants (NTI), defined daily doses (DDD) per 1000 inhabitants per day (DID), and DDD per 100 visits. Segmented regression analysis was applied to monthly prescriptions to assess the utilisation trends. **Results**: Antibiotic prescription rates increased significantly, from 3.5% in 2022 to 4.0% in 2023 (*p* = 0.001), with a 9.5% relative increase (38,920 prescriptions for 1,112,574 visits to 42,613 prescriptions for 1,063,646 visits). Respiratory conditions drove the increase in antibiotics use, with a 68.3% rise in prescriptions, with upper respiratory tract infections being the most common diagnosis for antibiotic prescriptions (n = 9296 prescriptions in 2023), with a steady monthly upward trend. Access group antibiotics accounted for >90% of prescriptions. The most antibiotics were prescribed for acne, with 36,304 DDD per 100 visits in 2023. Both NTI and DID significantly increased in 2022, largely contributed by a >100% increase in Watch group antibiotic use. Total antibiotic NTI dipped slightly in 2023, with a stable trend in both NTI and DID for all antibiotics. **Conclusions**: The post-COVID-19 pandemic surge in the antibiotic prescription rate for respiratory conditions and Watch group antibiotic use highlight the need for targeted stewardship interventions. Optimising acne treatment and diagnosis coding are key strategies to further reduce unnecessary prescriptions.

## 1. Introduction

As the global burden of antimicrobial resistance (AMR) continues to rise, with an estimated 4.71 million deaths associated with bacterial AMR in 2021, improving care could potentially prevent up to 92 million deaths worldwide between 2025 and 2050 [1]. Primary care remains the predominant source of antibiotic prescriptions for patients, accounting for majority of the antibiotics dispensed for common infections [2,3]. This contributes to the selection of drug-resistant bacteria, with increased resistance rates associated with longer treatment durations and multiple antibiotic courses [4].

Singapore experienced a significant decline in antibiotic prescriptions for both adults and children in primary care settings during 2020 and 2021, compared to the pre-pandemic years of 2018 and 2019 [5,6]. This improvement was noted in the use of Access group over Watch group antibiotics, as classified by the World Health Organization’s (WHO) AWaRe (Access, Watch, Reserve) framework. The reduction in antibiotic use was attributed to reduced outpatient visits, altered infectious disease presentations, and changes in prescribing practices, coupled with public health measures, including testing, mask use, and vaccination campaigns [4,7,8,9,10]. As the world recovers from the pandemic, assessing the post-pandemic impact on antibiotic use in Singapore’s primary care settings is crucial, given concerns that prescribing rates may revert to pre-pandemic levels, thereby exacerbating AMR. Globally, there have been reports of a rebound in acute respiratory infection rates post-COVID-19, accompanied by increased antibiotic prescriptions, a phenomenon worth investigating in Singapore as well [11].

Furthermore, the persistence of inadequate documentation of antibiotic use in patient records and inaccurate diagnosis coding beyond the pandemic period remains a concern, which is consistent with the findings from earlier studies. It is pertinent to detail that, in Singapore, the COVID-19 public health emergency measures were lifted in February 2023, with mask requirements lifted in all public places except in healthcare facilities [12]. Therefore, we aim to investigate the post-pandemic effects on antibiotic utilisation patterns in Singapore’s primary care setting, examining antibiotic utilisation rates among specific demographics and conditions, and assessing the quality and quantity of antibiotic use in 2022 and 2023. The findings can be used to inform evidence-based strategies for optimising antibiotic stewardship programmes within primary care.

## 2. Results

Antibiotic prescription rates significantly increased from 3.50% (38,920 prescriptions out of 1,112,574 visits) in 2022 to 4.01% (42,613 prescriptions out of 1,063,646 visits) in 2023 (*p* < 0.001), with a relative increase of 9.5% (Table 1). The median age of patients receiving antibiotics was 47.3 years, with over a quarter of antibiotics prescribed to those aged 21–44. Age-related differences in antibiotics prescribed were observed, with 6.0% of visits by patients aged 18–44 resulting in antibiotic prescriptions in 2023. Compared to male patients, female patients had a higher number of antibiotic prescriptions in both 2022 and 2023 (55.1% and 53.9%), with higher percentages of visits prescribed antibiotics (3.86% compared to 3.14% in 2022 and 4.28% compared to 3.73% in 2023). Differences in antibiotics received were also noted among different ethnic groups, with ethnically Indian patients having the highest percentage of visits receiving antibiotics in 2022 and 2023 (4.3% and 4.95%, respectively). Additionally, antibiotic prescription rates also varied across different practices and visit types (Table 1), with Clinic E having the highest percentage of visits prescribing antibiotics in 2022 and 2023 (4.2% and 5.46%, respectively).

**Figure 1:**
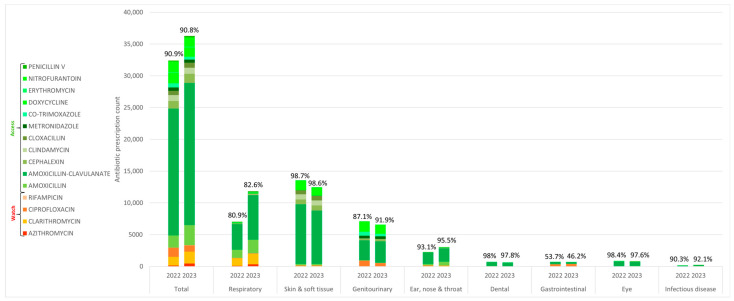
Oral antibiotics classified according to WHO AWaRe, appended with percentages of access group antibiotic use within condition groups, 2022–2023.

An analysis of antibiotic use quality revealed that Access group antibiotics constituted a consistently high proportion of prescribed antibiotics, exceeding 90% in both 2022 and 2023 (Figure 1). Conversely, Watch group antibiotic use exhibited an increase in prescriptions for gastrointestinal conditions, primarily driven by rising from 46.3% in 2022 to 53.8% in 2023. Amoxicillin–clavulanate remained the most frequently prescribed antibiotic in 2022 (61.5%, n = 19,832) and 2023 (61.7%, n = 42,071).

**Figure 2:**
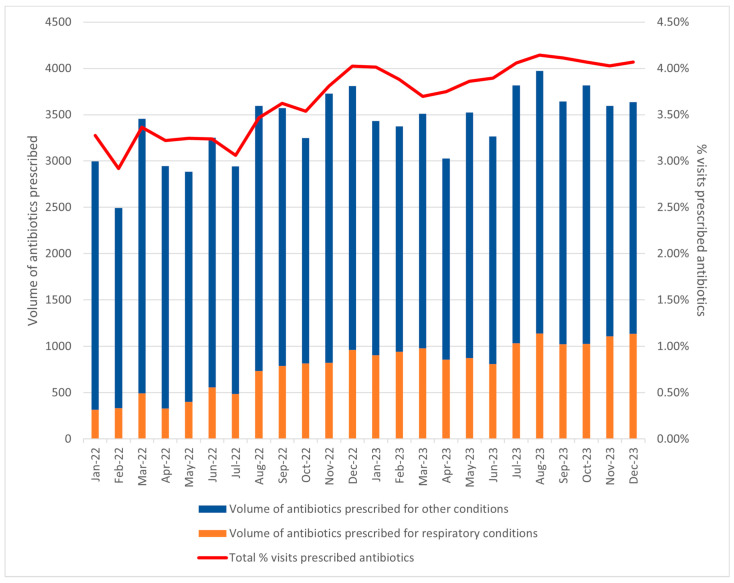
Trend of monthly volume of antibiotics prescribed for respiratory conditions in 2022–2023, and % visits prescribed antibiotics per month.

The increase in antibiotic use was primarily driven by a 68.3% increase in antibiotics prescribed for respiratory conditions (Table 2). In 2022, respiratory conditions accounted for 18.1% of the total antibiotics prescribed (n = 7034), with 3.36% of visits resulting in antibiotic prescriptions. In contrast, 2023 saw a notable surge, with 11,834 prescriptions, constituting 27.8% of all antibiotics prescribed, and 5.46% of visits prescribed antibiotics. From January 2022 to December 2023, an uptrend in antibiotic prescriptions for respiratory conditions was observed (Figure 2). Concurrently, the antibiotic prescription rate rose from 3.28% in January 2022 to above 4% from July 2023 onwards, also indicating a higher proportion of visits resulting in antibiotic prescriptions.

Respiratory conditions have become the second most prevalent reason for antibiotic use in 2023, accounting for 27.8% of all antibiotic prescriptions, with upper respiratory tract infection (URTI) being the most common diagnosis (n = 9296 prescriptions), with 5.3% of URTI visits prescribed antibiotics. In addition, antibiotic prescriptions for pneumonia increased by more than 2.5 times, with 59.4% of visits prescribed antibiotics. Other respiratory conditions that had high percentages of visits prescribed antibiotics in 2023 included acute bronchitis (n = 199 prescriptions, 25.9% of visits prescribed antibiotics), bronchiectasis (n = 69 prescriptions, 25.8% of visits prescribed antibiotics), and chronic obstructive pulmonary disease with acute exacerbation (n = 59 prescriptions, 28.0% of visits prescribed antibiotics). Antibiotics prescribed for respiratory conditions surpassed genitourinary conditions in 2023, which accounted for 15.5% of all antibiotics prescribed, and closely trailed behind skin and soft-tissue conditions (29.3%) in 2023 (Table 2). Additionally, eyelid and dental conditions, which typically do not require antibiotics, had high percentages of visits prescribed antibiotics, at 13.1% and 12.7%, respectively. However, limitations in diagnostic coding accuracy hindered the determination of the true cause of antibiotic prescriptions in 2071 cases (2.54%) (grouped under multiple diagnoses in Table 2), while 10,782 prescriptions (13.2%) lacked infection-related diagnosis codes despite antibiotic prescriptions (labelled as undefined in Table 2).

An analysis of the quantity of antibiotics prescribed revealed notable trends. Acne topped the list of diagnoses with the highest antibiotic usage in 2023, with a substantial 36,304 DDD per 100 visits. Unspecified skin disorders treated with metronidazole followed closely, accounting for 19,850 DDD per 100 visits. There was an increase of 0.56 (21.33%) in the NTI and 0.55 (53.51%) in the DID for all antibiotics, with a statistically significant positive average monthly change in NTI of 0.03 (95% CI 0.015–0.045) (Table 3). Monthly dispensing of Access group antibiotics’ NTI showed a significant increase (*p* < 0.001). In contrast, the average monthly change in DID was not statistically significant (*p* = 0.231). Conversely, Watch group antibiotic prescriptions saw a substantial increase, with relative changes of 101.54% in NTI and 174.72% in DID. This was accompanied by a statistically significant positive average monthly change for both NTI (*p* = 0.009) and DID (*p* = 0.014), indicating a considerable rise in both prescription volume and antibiotic quantity for Watch group antibiotics, impacting total antibiotic prescriptions.

Our segmented regression analyses showed that, in 2022, there was a significant upward trend in both NTI (β_1_ = 0.07, 95% CI 0.03–0.11, *p* = 0.001) and DID (β_1_ = 0.11, 95% CI 0.003–0.21, *p* = 0.045) (Table 4). However, in 2023, there is a statistically significant decline in NTI, with a level change (β_2_) of −0.39 (95% CI −0.78–−0.007, *p* = 0.05), and a non-significant decline in DID, with a level change (β_2_)of −0.59 (95% CI −1.63–−0.44, *p* = 0.25). The trend change (β_3_) was not significant for both NTI and DID in 2023 (*p* = 0.17 and 0.18 respectively.) For watch group antibiotics, the average NTI and DID significantly increased (β_1_, *p* < 0.001 for both) but showed a significant immediate decrease (β_2_, *p* < 0.001 for both) and slowing down of the rate of increase (β_3_, *p* = 0.02 and 0.01 respectively).

## 3. Discussion

The COVID-19 pandemic has introduced unprecedented challenges in primary care, such as logistical demands in vaccine management, the establishment of community clinics for the diagnosis and management of patients with respiratory tract infections, and the implementation of enhanced infection control measures [13]. As these pandemic-related services stood down post-pandemic, our analysis revealed a disturbing trend: a significant 9.5% increase in antibiotic utilisation in primary care, reversing the progress made in reducing antibiotic use in previous years [5,6,14]. Initially, in our previous study, we observed a steep decline (January–May 2020) when the pandemic first hit Singapore’s shores, followed by a period of relative stability, with low variations from May 2020 to December 2021. From 2022 onwards, antibiotic prescription rates for respiratory conditions have been steadily increasing at a slower rate compared to the initial decline, returning to pre-pandemic volumes from July 2023 onwards. Of note, in pre-pandemic times, typical fluctuations in visits prescribed antibiotics at 1% were similar compared to our current study findings post-pandemic, showing that the magnitudes of monthly changes did not seem to be changed due to the COVID-19 pandemic.

In 2022–2023, Access group antibiotic use remained consistently high at 90%, surpassing the WHO’s benchmark of 60% and aligning with the standards of other countries, which typically ranged between 50 and 85% [15,16]. This may have been attributed to the restricted formulary options available in primary care clinics, which may have contributed to more judicious antibiotic prescribing practices. Moreover, antibiotics were not able to be purchased over the counter, unlike in many Southeast Asian countries. This requirement for a legal prescription before antibiotic use also helped to improve our antibiotic use. Our segmented regression analysis showed significant positive average monthly changes in both the NTI and DID, indicating an increase in watch group antibiotic prescriptions and use in 2022. But, the trends remained reassuring in 2023, where rates of increase have slowed and stabilised, suggesting a potential plateauing. Notably, the larger increases in DID (53.51%) compared to NTI (21.33%) suggest that, not only were antibiotics being prescribed more, but the dose and duration may also be increasing.

The surge may be attributed to several factors, including the resurgence of respiratory infection presentations, increased Watch group antibiotic use for gastrointestinal conditions, reduced COVID-19 testing by physicians and patients, a relaxation of social distancing, hygiene practices, and border re-openings [17,18]. Furthermore, the shift of patients from emergency departments, telehealth, and private care to primary care may increase the complexity of cases and ambiguity in diagnosis [19]. While Singapore has demonstrated lower antibiotic prescribing rates for inpatients compared to other countries in Southeast Asia [20], outpatient antibiotic use remains a concern, with patterns similar to neighbouring countries like Malaysia [21].

Our study showed demographic and ethnic differences in antibiotic use, which we postulated was due to differences in presentations of common infections across the life cycle and socio-demographically driven health beliefs shaped by cultural practices, education, and socioeconomic status [22,23]. Acne, the leading condition for antibiotic prescriptions (in DDD), predominantly affects adolescents and young adults, with high rates of oral antibiotic use [24]. Furthermore, Singapore’s tropical climate, characterised by high temperatures and humidity, may exacerbate acne severity and prevalence, as these environmental factors can trigger acne flares [25]. Younger patients with acute conditions had higher antibiotic prescription rates, due to differing healthcare utilisation patterns in public primary care, where elderly patients attend mainly for chronic care and younger patients for acute conditions [26]. Moreover, local guidelines suggest a combination of oral antibiotics with acne-related medications as the first line of treatment for moderate-to-severe acne, which also may result in higher oral antibiotic use [27]. This could also be attributed to inadequate knowledge and misconceptions about antibiotic use, compounded by a lack of continuity of care and trust-building with a regular doctor [28]. This is reinforced by our study findings, where two-thirds of antibiotics were actually prescribed during acute care visits, highlighting a potential target for optimising antibiotic use.

Respiratory conditions fuelled a staggering 68.3% surge in antibiotic use, driven by increased URTI consultations and antibiotic prescriptions. A possible explanation could be the changing pattern of circulating respiratory viruses in Singapore post-pandemic, altering the typical seasonal patterns of influenza and RSV, with peak influenza activity occurring 3–8 weeks later than pre-pandemic times [29]. However, our study suggested that the increase in antibiotic use cannot be solely attributed to the increase in URTI visits. Instead, we observed an increase in antibiotics prescribed per URTI visit from 3.25% in 2022 to 5.30% in 2023. This increase in the antibiotic prescription rate for URTI may be attributed to various factors, including shifting perceptions of antibiotic use among primary care physicians, changes in patient awareness, and shared decision-making dynamics [30]. In addition, increasing practice volume, time pressures, and financial considerations may have contributed to this [31]. Future studies could delve into the perspectives of patients and physicians on antibiotic use in the post-pandemic era to uncover the underlying factors driving antibiotic prescribing for URTI. In our study, conditions typically considered viral in nature, such as COPD exacerbations and acute bronchitis, showed alarmingly high antibiotic prescription rates. This suggests potential knowledge gaps among patients and physicians, patient-driven requests, or challenges to accurately diagnosing bacterial versus viral causes [32,33]. The COVID-19 pandemic may have shifted knowledge and attitudes; further research is necessary to reassess these factors. Evidence-based strategies, including auditing and feedback to benchmark prescribing practices, and rapid diagnostic tools to differentiate bacterial from viral infections have demonstrated effectiveness in combatting excessive antibiotic prescribing, which can be tailored and implemented within local settings to inform programmes to promote judicious use [34,35,36].

A comparison with our previous study revealed a post-pandemic decline in antibiotics coded for non-infectious diagnoses, with the current trends indicating stable antibiotic use, suggesting heightened physician awareness and enhanced coding practices, possibly due to pandemic-related experiences. Additionally, the waning pandemic may have freed up resources, enabling greater focus on antibiotic stewardship initiatives. For example, Singapore has strengthened efforts to optimise antibiotic use, driven by the Ministry of Health’s (MOH) Agency of Care Effectiveness (ACE) and the Antimicrobial Resistance Coordinating Office (AMRCO). A key milestone is the introduction of ACE clinical guidance on urinary tract infections in 2023 [37]. This is complemented by a collaborative approach between private general practitioners (GPs) and AMRCO through the GP–Antimicrobial Utilisation Surveillance Initiative, which monitors and provides feedback on utilisation trends. Both endeavours align with the National Strategic Action Plan (NSAP) on AMR, launched in 2017, and pave the way for a national ASP strategy in primary care [38].

### Strengths and Limitations

Our study boasted a few methodological and analytical strengths, providing a comprehensive evaluation of antibiotic use in primary care. By leveraging a 48-month longitudinal design, extensive data coverage from all public primary care clinics in Western Singapore, and standardised calculations for NTI and DID, we ensured comparability with other studies conducted internationally. Utilising segmented regression analysis with robust data and no missing values significantly enhanced the reliability and validity of our findings. Our study also used the AWaRe classification as a marker of antibiotic quality, a key metric recommended by the WHO and featured as a national target in many countries. The patient-level data analysis enabled accurate identification of target patient demographics and infections for future interventions. Ultimately, our research contributed significantly to the global discourse on antibiotic use, informing public health policy and providing valuable insights for targeted interventions and the planning of effective AMR strategies.

Our study has several limitations. Our trend models lacked covariates for patient demographics and clinical factors, potentially introducing residual confounding that may affect the interpretation of our findings. The inclusion of children in DDD calculations may overestimate antibiotic use due to the standardised adult dosages being higher than the actual pediatric dosages. We were unable to distinguish between antibiotic use for acute conditions versus those prescribed for prophylaxis, although these prescriptions were relatively rare. The AWaRe classification, while useful, is only one measure of prescribing quality and may not accurately reflect actual clinic needs or the appropriateness of prescribing according to the guidelines, which was not feasible to assess due to a lack of patient case notes. Additionally, our study’s focus on public primary care clinics in Singapore may not be representative of private general practices, who may prescribe more antibiotics, as they see a younger population with a lesser chronic disease burden. Furthermore, this study relied on electronic health records data, assuming accurate data extraction, and was limited by its retrospective design and potential biases in antibiotic prescribing practices. Lastly, the prescribed data may not equate to the dispensed data, which could impact our findings.

## 4. Methods

### 4.1. Data Source and Study Population

This retrospective observational study utilised data extracted from the electronic medical record database (Epic Clarity) used in seven Singapore public primary care clinics, from 1 January 2022 to 31 December 2023. The seven clinics were part of the National University Health System (NUHS) cluster, one of Singapore’s three healthcare clusters, serving a population of approximately 1.14 million residents in Western Singapore [13,39]. The study included patients of all ages who had visited one of these seven clinics and were prescribed an oral antibiotic during the same visit. De-identification was performed by a centralised, trusted third party (institution research office) before handing to the study team for analysis.

The variables extracted from Epic included patient demographics such as age, gender, race, clinic visited, visit type, coded visit diagnoses [using the International Classification of Diseases 10th revision (ICD-10)], and antibiotic name, dose, duration, and frequency. We included all oral antibiotics available within our drug formulary in this study: penicillins (Amoxicillin, Amoxicillin-clavulanate, Cloxacillin, and Penicillin V), macrolides (Azithromycin, Clarithromycin, and Erythromycin), tetracyclines (Doxycycline), fluoroquinolones (Ciprofloxacin), cephalosporins (Cephalexin), lincosamides (Clindamycin), sulfonamides (Co-trimoxazole), nitrofurans (Nitrofurantoin), anti-tubercular agents (Rifampicin), and imidazoles (Metronidazole). Institutional-level data on the total number of visits for each category were also obtained from existing data to facilitate a comprehensive analysis. These data enabled the determination of the breakdown of antibiotics prescribed for each condition, as well as the calculation of antibiotic prescription rates stratified by individual categories, including segmentation by month, age, sex, race, and other relevant factors. To ensure data accuracy, data quality checks were performed through a manual review of a random sample of medical records. Leveraging our established methodology and extraction techniques, refined through previous large-scale data extractions, we were able to achieve comprehensive data capture, resulting in a complete dataset with no missing values.

### 4.2. Determining Quality of Antibiotic Use: Diagnosis Categorisation and AWaRE Classification

For quality of antibiotic use, we used the 2021 WHO AWaRe classification, categorising them by AMR risk: Access (low risk, for use as first or second line), Watch (moderate risk, restricted use), and Reserve (high risk, last resort) group antibiotics. A minimum target of 60% of access group antibiotics was used as the benchmark for quality, in accordance with WHO standards [40].

To visualise the usage of antibiotics, we categorised visits with oral antibiotics into seven groups based on indicated diagnoses: respiratory, skin and soft tissue, genitourinary, gastrointestinal, infectious disease, dental, and undefined (miscellaneous or chronic disease diagnoses with unclear indications) (Table S1). Our classification of conditions requiring or not requiring antibiotics was based on our previous methodology, which adhered to the diagnosis codes established by WHO’s ICD-10 [5,6]. For visits with multiple diagnoses, a tiered ranking logic system prioritised infective conditions over non-infective ones, ensuring that each prescription belonged to only one category. Prescriptions with unclear indications due to multiple non-infective diagnoses were categorised as ‘multiple diagnoses’. All antibiotics prescribed by dentists were presumed to be for the treatment of dental conditions. We further analysed the antibiotics prescribed in conjunction with infective visit diagnoses to determine the antibiotic prescribing burden associated with specific infective conditions and incorporated these specific diagnoses chosen by physicians within the condition groups.

### 4.3. Determining Quantity of Antibiotic Use: NTI, DID, and Heatmap Visualisation

To quantity antibiotic use, we used two widely recognised metrics: monthly number of items dispensed/1000 inhabitants (NTI) and monthly defined daily doses (DDDs)/1000 inhabitants/day (DID), a methodology employed in numerous international studies [41,42,43]. For NTI, we calculated the total monthly number of antibiotic items dispensed for each group (Access, Watch, and total), divided by the total population of Western Singapore, and multiplied by 1000. To calculate DID, we used the World Health Organization’s (WHO) DDD definition, which represents the average maintenance dose for adults. We multiplied the total monthly quantity dispensed by the antibiotic strength, divided the result by the WHO-defined DDD value, and then, divided the result by the population residing in Western Singapore, multiplied by 1000, and divided by the number of days in the month [39]. We acknowledge that DDDs will underestimate antibiotic usage in children, as paediatric dosing is typically weight-based rather than standardised to a fixed adult dose. To assess the overall burden of antibiotic use, we calculated DDD per 100 visits, providing a standardised measure of antibiotic utilisation for specific diagnoses.

To further explore trends in antibiotic use for respiratory conditions, we analysed the monthly antibiotic prescribing volume in relation to respiratory infection visits. A time-series plot was created to visualise the trend of monthly antibiotic prescriptions for respiratory conditions in 2022 and 2023, allowing us to assess whether antibiotic use increased disproportionately to the number of respiratory infection visits.

### 4.4. Statistical Analysis

IBM SPSS Statistics Version 25.0, R Version 4.2.0, and Microsoft Excel 2010 were used in the cleaning and analysis. Descriptive statistics were performed, with numerical variables being represented as mean with standard deviations (SD) for normally distributed continuous variables, median with inter-quartile range (IQR) for non-normal continuous variables, and n (%) for categorical variables. The antibiotic prescription rate was calculated as a proportion of the total number of patient visits, with the numerator representing the number of antibiotic prescriptions and the denominator representing the total number of visits. This rate was further stratified and analysed by various demographic and clinical variables, including age, gender, race, polyclinic location, and visit type, to provide a detailed understanding of antibiotic prescribing patterns. To describe changes in antibiotic prescription utilisation trends between 2022 and 2023, we calculated three metrics to evaluate the changes in NTI/DID over time. The absolute change represents the difference in NTI/DID between the first and last month of the dataset. The relative change expresses this difference as a percentage. A trend analysis employing linear regression was conducted to determine the average monthly change in utilisation from 2022 to 2023. To verify the assumptions of our linear regression model, we plotted the standardised residuals against the standardised predicted values, confirming a normal distribution with constant variance. We also checked for outliers, finding standardised residual values within the range of −1.95 to 1.54, and conducted a Durbin–Watson test, which resulted in a value of 2.14, supporting the assumption of independence. Lastly, a segmented regression analysis of interrupted time series was performed to assess the changes between 2022 and 2023. The results, in terms of the regression coefficients and corresponding 95% confidence intervals (CI) and *p*-values, were presented (β_1_, β_2_, and β_3_).

## 5. Conclusions

The post-COVID-19 pandemic surge in antibiotic prescriptions for respiratory conditions and watch group antibiotic use highlights the need for targeted stewardship interventions, such as implementing and enforcing adherence to guidelines, physician audit and feedback, and using rapid diagnostic tests to improve diagnosis and antibiotic therapy. Furthermore, optimising antibiotic use in acne could significantly reduce the volume of antibiotics prescribed, especially among younger patients who often receive prolonged courses for chronic acne management. Accurate diagnosis code labelling is also crucial for enabling targeted pharmacy interventions within a comprehensive national AMS strategy in primary care, ultimately promoting responsible antibiotic use and mitigating AMR.

## Figures and Tables

**Table 1:** Oral antibiotic prescriptions in primary care, 2022–2023.

	2022		2023	
	N (%)	Prescription Rate, %	N (%)	Prescription Rate, %
Total prescriptions	38,920 (100.0)	3.50	42,613 (100.0)	4.01
Age, Mean (SD)	47.2 (22.4)	-	47.3 (23.3)	-
Age, Median (IQR)	50 (38)	-	51 (39)	-
Age group				
0–17	3147 (8.1)	2.35	4604 (10.8)	3.55
18–20	1791 (4.6)	4.90	1696 (4.0)	6.30
21–44	12,356 (31.7)	4.99	12,217 (28.7)	5.91
45–54	5121 (13.2)	3.71	5265 (12.4)	4.13
55–64	6376 (16.4)	3.01	7089 (16.6)	3.44
65–74	6220 (16.0)	2.85	6865 (16.1)	3.05
≥75	3909 (10.0)	3.09	4877 (11.4)	3.45
Gender				
Male	17,476 (44.9)	3.14	19,631 (46.1)	3.73
Female	21,444 (55.1)	3.86	22,982 (53.9)	4.28
Race				
Chinese	26,000 (66.8)	3.39	28,773 (67.5)	3.83
Malay	5979 (15.4)	3.36	6373 (15.0)	4.09
Indian	4189 (10.8)	4.30	4534 (10.6)	4.95
Others	2752 (7.07)	3.87	2933 (6.88)	4.50
Polyclinic				
Clinic A	6733 (17.3)	3.43	7803 (18.3)	4.01
Clinic B	5124 (13.2)	3.05	4423 (10.4)	3.26
Clinic C	7092 (18.2)	4.09	6527 (15.3)	4.33
Clinic D	5626 (14.5)	3.19	6710 (15.7)	3.51
Clinic E	7122 (18.3)	4.20	9199 (21.6)	5.46
Clinic F	4284 (11.0)	3.51	4071 (9.55)	3.54
Clinic G	2939 (7.55)	2.76	3880 (9.11)	3.60
Visit Type				
Acute	25,645 (65.9)	5.75	28,634 (67.2)	7.29
Chronic	13,275 (34.1)	1.99	13,981 (32.8)	2.08

**Table 2:** Condition groups and diagnoses prescribed antibiotics, 2022–2023.

Conditions	2022		2023		Total	
	No of Antibiotic Prescriptions, (%)	% Visits Prescribed Antibiotics	No of Antibiotic Prescriptions, (%)	% Visits Prescribed Antibiotics	No of Antibiotic Prescriptions, (%)	% Visits Prescribed Antibiotics
Skin and Soft Tissue	13,562 (34.8%)	11.3	12,471 (29.3%)	10.4	26,033 (31.9%)	10.8
Disorder of skin and subcutaneous tissues	4058 (50.4%)	9.88	3118 (44%)	9.02	7176 (47.4%)	9.49
Cellulitis	2812 (34.9%)	73.9	2885 (40.7%)	71.3	5697 (37.6%)	72.5
Abscess	1179 (14.7%)	48.0	1083 (15.3%)	47.7	2262 (15%)	47.9
Respiratory	7034 (18.1%)	3.36	11,834 (27.8%)	5.46	18,868 (23.1%)	4.42
Acute upper respiratory tract infection	5650 (79.1%)	3.25	9296 (77.0%)	5.30	14,946 (77.8%)	4.28
Pneumonia	365 (5.11%)	53.2	977 (8.09%)	59.4	1342 (6.98%)	57.6
Disorder of respiratory system	391 (5.47%)	12.1	590 (4.89%)	17.6	981 (5.11%)	14.9
Genitourinary	7094 (18.2%)	29.7	6588 (15.5%)	29.7	13,682 (16.8%)	29.7
Urinary tract infection	6007 (94.3%)	78.8	5526 (94.2%)	76.1	11,533 (94.3%)	77.4
Disorder of female genital organs	223 (3.5%)	12.2	159 (2.71%)	11.6	382 (3.12%)	12.0
Urinary disorder	142 (2.23%)	15.3	180 (3.07%)	14.1	322 (2.63%)	14.6
Ear, Nose, and Throat	2272 (5.84%)	8.83	3046 (7.15%)	10.7	5318 (6.52%)	9.81
Disorder of ear	659 (52.9%)	12.3	619 (36.8%)	12.3	1278 (43.6%)	12.3
Acute tonsillitis	256 (20.5%)	83.1	686 (40.8%)	81.2	942 (32.2%)	81.7
Otitis media	332 (26.6%)	58.2	378 (22.5%)	55.8	710 (24.2%)	56.9
Eye	853 (2.19%)	2.65	804 (1.9%)	2.53	1657 (2.03%)	2.59
Disorder of eyelid	452 (66.7%)	13.3	341 (56.6%)	13.1	793 (61.9%)	13.2
Disorder of eye	183 (27%)	1.13	129 (21.4%)	0.832	312 (24.4%)	0.986
Conjunctivitis	43 (6.34%)	1.68	133 (22.1%)	3.51	176 (13.7%)	2.77
Gastrointestinal	724 (1.86%)	0.737	699 (1.64%)	0.865	1423 (1.75%)	0.795
Gastroenteritis, acute	315 (58.3%)	1.04	354 (67.3%)	1.49	669 (62.8%)	1.24
Abdominal pain	172 (31.9%)	2.53	120 (22.8%)	2.29	292 (27.4%)	2.42
Anorectal abscess	53 (9.81%)	62.4	52 (9.89%)	64.2	105 (9.85%)	63.3
Dental	706 (1.81%)	12.7	623 (1.46%)	12.2	1329 (1.63%)	12.5
Infectious diseases	154 (0.396%)	1.03	216 (0.507%)	2.17	370 (0.454%)	1.48
Multiple Diagnoses	827 (2.12%)	-	1244 (2.92%)	-	2071 (2.54%)	-
Undefined	5694 (14.6%)	-	5088 (11.9%)	-	10,782 (13.2%)	-

**Table 3:** Absolute, relative, and average monthly changes for the number of access, watch and total antibiotics dispensed/1000 inhabitants, defined daily doses/1000 inhabitants/day, 2022–2023.

	Absolute Change	Relative Change	Average Monthly Change (95% CI)	*p*
Number of antibiotics dispensed/1000 inhabitants (NTI)				
Total	0.56	21.33%	0.030 (0.015, 0.045)	<0.001
Access	0.39	15.74%	0.026 (0.013, 0.039)	<0.001
Watch	0.17	101.54%	0.004 (0.001, 0.007)	0.009
Number of Defined Daily Doses (DDD)/1000 inhabitants/day (DID)				
Total	0.55	53.51%	0.024 (−0.014, 0.062)	0.206
Access	0.48	48.72%	0.022 (−0.015, 0.060)	0.231
Watch	0.068	174.72%	0.001 (0.0003, 0.003)	0.014

Data are presented as regression coefficients (95% CI) and *p*-value, calculated using linear regression analysis.

**Table 4:** Trend analyses using segmented regression on antibiotic use trends in 2022–2023.

	Baseline Trend in 2022 (β_1_)		Level Change in 2023 (β_2_)		Trend Change in 2023 (β_3_)	
Number of antibiotics dispensed/1000 inhabitants (NTI)						
	B (95% CI)	*p*	B (95% CI)	*p*	B (95% CI)	*p*
Total	0.07 (0.03–0.11)	0.001	−0.39 (−0.78–−0.007)	0.05	−0.04 (−0.09–0.02)	0.17
Access	0.06 (0.02–0.09)	0.006	−0.26 (−0.62–0.1)	0.15	−0.03 (−0.08–0.03)	0.29
Watch	0.02 (0.01–0.02)	<0.001	−0.13 (−0.19–−0.07)	<0.001	−0.01 (−0.02–−0.002)	0.02
Number of Defined Daily Doses (DDD)/1000 inhabitants/day (DID)						
	B (95% CI)	*p*	B (95% CI)	*p*	B (95% CI)	*p*
Total	0.11 (0.003–0.21)	0.045	−0.59 (−1.63–0.44)	0.25	−0.1 (−0.25–0.05)	0.18
Access	0.1 (−0.004–0.21)	0.06	−0.55 (−1.59–0.49)	0.28	−0.1 (−0.25–0.05)	0.2
Watch	0.006 (0.004–0.008)	<0.001	0.04 (−0.07–−0.02)	<0.001	−0.004 (−0.007–−0.001)	0.01

Data are presented as regression coefficients (B), 95% confidence intervals (95% CI) and *p*-value (*p*).

## Data Availability

The data presented in the study are available on request from the corresponding author.

## Supplementary Materials

The following supporting information can be downloaded at https://www.mdpi.com/article/10.3390/antibiotics14030309/s1: Table S1: List of Diagnostic Codes and Classifications.
