# Supplementary material for: Increase in Antibiotic Utilisation in Primary Care Post COVID-19 Pandemic
Source: Antibiotics (Basel). 2025 Mar 17;14(3):309. doi: 10.3390/antibiotics14030309 (PMC11939422; doi:10.3390/antibiotics14030309)
Supplement: Supplementary file 1 [file antibiotics-14-00309-s001.zip › antibiotics-3537164-supplementary.pdf]

Table S1. List of Diagnostic Codes and Classifications

Respiratory Conditions (Presumed To Be Infective)							
RESPIRATORY TUBERCULOSIS UNSPECIFIED, WITHOUT MENTION OF BACTERIOLOGICAL OR HISTOLOGICAL CONFIRMATION	TUBERCULOSIS	PNEUMONIA	PNEUMONIA, UNSPECIFIED	COPD	CHRONIC OBSTRUCTIVE PULMONARY DISEASE, UNSPECIFIED	CHRONIC OBSTRUCTIVE PULMONARY DISEASE (COPD)	CAP (COMMUNITY ACQUIRED PNEUMONIA)
PULMONARY TUBERCULOSIS	ASTHMA-COPD OVERLAP SYNDROME	BRONCHIECTASIS	WHOOPING COUGH, UNSPECIFIED	COPD WITH ACUTE EXACERBATION	PERTUSSIS	MYCOPLASMA INFECTION	ACUTE BRONCHIOLITIS
**Respiratory Conditions (Presumed To Be Non-Infective)**							
ACUTE BRONCHITIS, UNSPECIFIED	ACUTE UPPER RESPIRATORY INFECTION, UNSPECIFIED	ASTHMA	ASTHMA, UNSPECIFIED	INFLUENZA WITH OTHER RESPIRATORY MANIFESTATIONS, INFLUENZA VIRUS IDENTIFIED	UPPER RESPIRATORY TRACT INFECTION	URTI (ACUTE UPPER RESPIRATORY INFECTION)	COVID-2019: SUSPECT CASE
URTI	INFLUENZA-LIKE ILLNESS	ACUTE BRONCHITIS	DISORDER OF RESPIRATORY SYSTEM	RESPIRATORY DISORDER, UNSPECIFIED	OTHER RESPIRATORY CONDITIONS	ASTHMA(BRONCHIAL)	CORONAVIRUS INFECTION, UNSPECIFIED SITE

PULMONARY EMBOLISM WITHOUT MENTION OF ACUTE COR PULMONALE	PULMONARY EMBOLISM	CORONAVIRUS INFECTION	RESPIRATION DISORDER	COUGH	CHRONIC COUGH	UPPER AIRWAY COUGH SYNDROME	HAEMOPTYSIS
ABNORMAL CXR (CHEST X-RAY)	ABNORMAL FINDINGS ON DIAGNOSTIC IMAGING OF LUNG	PLEURAL EFFUSION	HERPANGINA				
**Skin Conditions (Presumed To Be Infective)**							
ACNE, UNSPECIFIED	ABSCESS	CARBUNCLE OF SKIN AND/OR SUBCUTANEOUS TISSUE	CELLULITIS	CELLULITIS, UNSPECIFIED	BURN OF UNSPECIFIED BODY REGION, UNSPECIFIED THICKNESS	DISORDER OF NAIL	UNSPECIFIED DIABETES MELLITUS WITH FOOT ULCER DUE TO MULTIPLE CAUSES
FURUNCLE OF SKIN OR SUBCUTANEOUS TISSUE	NAIL DISORDER, UNSPECIFIED	DM FOOT	OPEN WOUND OF UNSPECIFIED BODY REGION	ULCER OF LOWER LIMB, NOT ELSEWHERE CLASSIFIED	BURNS	SKIN INFECTION	OPEN WOUND
DIABETIC FOOT ULCER	NAIL DISEASE	CHRONIC ULCER OF LOWER EXTREMITY	ACNE	FB SKIN	MULTIPLE WOUNDS	CUTANEOUS ABSCESS, FURUNCLE AND CARBUNCLE, UNSPECIFIED	DECUBITUS ULCER AND PRESSURE AREA, UNSPECIFIED

INJURY	INJURY, UNSPECIFIED	SUPERFICIAL FOREIGN BODY (SPLINTER) OF UNSPECIFIED BODY REGION	OTHER INJURIES	OTHER BREAST CONDITIONS	BURN	WOUND CELLULITIS	PRESSURE ULCER
SUPERFICIAL BURN	BOIL	FURUNCLE	PARONYCHIA OF LEFT THUMB	LACERATION	DOG BITE	CAT BITE	SKIN ABSCESS
FOLLICULITIS	FOREIGN BODY (FB) IN SOFT TISSUE	MASTITIS	PARONYCHIA OF GREAT TOE OF LEFT FOOT	PARONYCHIA OF FINGER	ACUTE MASTITIS	PARONYCHIA OF THIRD TOE OF RIGHT FOOT	CELLULITIS OF FOOT, RIGHT
BREAST ABSCESS	ERYSIPELAS	FOOT ULCER DUE TO SECONDARY DM	SURGICAL WOUND BREAKDOWN	SUPERFICIAL FOREIGN BODY	PARONYCHIA OF TOE	OSTEOMYELITIS	LOCALISED SKIN MASS, LUMP, OR SWELLING
LOCAL INFECTION OF SKIN AND SUBCUTANEOUS TISSUE	ENLARGED LYMPH NODE	PARONYCHIA OF FINGER OF RIGHT HAND	PARONYCHIA OF TOE OF RIGHT FOOT	POSTOPERATIV E WOUND INFECTION	INGROWN TOENAIL	INSECT BITE	LYMPHOEDEMA
PARONYCHIA OF FINGER OF LEFT HAND	PARONYCHIA OF TOE OF LEFT FOOT	PARONYCHIA OF RIGHT THUMB	IMPETIGO	BITE WOUND	SEPTIC ARTHRITIS	CHRONIC ULCER OF SKIN	GANGRENE
BULLOUS PEMPHIGOID	HAND DERMATITIS	DIABETIC ULCER OF LEFT FOOT	INFECTION OF ARTERIOVENOUS DIALYSIS FISTULA	UMBILICAL DISCHARGE	POSTOPERATIVE WOUND ABSCESS	INFECTIVE ARTHRITIS	BITE WOUND FROM MAMMAL
ROSACEA	HIDRADENITIS SUPPURATIVA	ENLARGED LYMPH NODES	FOREIGN BODY OF FINGER	LYMPHANGITIS	FOREIGN BODY IN FOOT	DIABETIC DERMOPATHY	FOREIGN BODY IN SKIN OR SUBCUTANEOUS TISSUE

BITTEN BY RAT	WOUND DUE TO RAT BITE	PILONIDAL CYST	CHRONIC OSTEOMYELITIS	FOREIGN BODY IN RIGHT FOOT	FOREIGN BODY IN HAND	FOREIGN BODY OF LEFT HAND	FOREIGN BODY OF RIGHT HAND
FOREIGN BODY IN LEFT FOOT							
**Skin Conditions (Presumed To Be Non-Infective)**							
SKIN DISORDER	DERMATOMYCOSIS	DISORDER OF SKIN AND SUBCUTANEOUS TISSUE	DISORDER OF SKIN AND SUBCUTANEOUS TISSUE, UNSPECIFIED	FLEXURAL ATOPIC DERMATITIS	FUNGAL INFECTION	NONSCARRING HAIR LOSS, UNSPECIFIED	OTHER ATOPIC DERMATITIS
OTHER PSORIASIS	SCABIES	SUPERFICIAL MYCOSIS, UNSPECIFIED	UNSPECIFIED CONTACT DERMATITIS, UNSPECIFIED CAUSE	VIRAL WARTS	CONTUSION	WARTS	ECZEMA
ABRASION	PRURITUS	PSORIASIS	CORN/CALLUS	URTICARIA	OTHER SKIN CONDITIONS	ALOPECIA	NEONATAL JAUNDICE
CORNS AND CALLOSITIES	OTHER SPECIFIED SOFT TISSUE DISORDERS, SITE UNSPECIFIED	SOFT TISSUE DISORDER	VARICOSE VEINS OF LOWER EXTREMITIES WITHOUT ULCER OR INFLAMMATION	VARICOSE VEINS, LEGS	DERMATITIS	ATOPIC DERMATITIS	NEONATAL JAUNDICE, UNSPECIFIED
CONTACT DERMATITIS	SKIN ABNORMALITIES	SEBACEOUS CYST	VARICOSE VEINS OF LOWER EXTREMITY	VIRAL WART	TINEA PEDIS	CORN	ASTEATOTIC ECZEMA
LIPOMA	CALLUS	SKIN TAG	ABRASION OF HEEL	CALLUS OF HAND	SQUAMOUS CELL CARCINOMA OF SKIN	RASH	MELANOCYTIC NAEVI

TINEA CORPORIS	INGROWING TOENAIL	INGROWING LEFT GREAT TOENAIL	INGROWN LEFT BIG TOENAIL	IGTN (INGROWING TOE NAIL)	INGROWING RIGHT GREAT TOENAIL	INGROWN NAIL OF GREAT TOE	DISORDER OF SKIN
LUMP IN NECK	GRANULOMA OF SKIN	NECK MASS	FOLLOW-UP EXAMINATION AFTER SURGERY FOR OTHER CONDITIONS	ATHEROSCLER OTIC PVD WITH ULCERATION	TINEA UNGUIUM	GANGLION CYST	GANGLION, SITE UNSPECIFIED
SWELLING OF LEFT SIDE OF FACE	TINEA	FUNGAL INFECTION OF SKIN	SEBORRHOEIC DERMATITIS	HAEMATOMA	CYST OF SKIN AND SUBCUTANEOUS TISSUE	ONYCHOMYCOSIS	DISEASE OF SEBACEOUS GLANDS
TINEA VERSICOLOR	PITYRIASIS VERSICOLOR	STATUS POST BELOW-KNEE AMPUTATION OF BOTH LOWER EXTREMITIES	HISTORY OF RIGHT BELOW KNEE AMPUTATION	ERYTHEMA MULTIFORME	EFFUSION OF JOINT	PYOGENIC GRANULOMA	PITYRIASIS RUBRA PILARIS
BRUISE	PARASITIC INFESTATION	JOINT SWELLING	MOLLUSCUM CONTAGIOSUM INFECTION	DERMATOPHYT OSIS	PITYRIASIS ROSEA	STATUS POST ABOVE-KNEE AMPUTATION OF BOTH LOWER EXTREMITIES	MOTOR VEHICLE ACCIDENT
MASTALGIA	DISORDER OF LACTATION	FIBROMA	SEBORRHOEA CAPITIS	STATUS POST AMPUTATION OF TOE	DISCOID LUPUS ERYTHEMATOS US	DISORDER OF BURSA	LIPODYSTROPHY
LICHEN PLANUS	PSORIASIS WITH ARTHROPATHY	DIAPER RASH	STATUS POST AMPUTATION OF FOOT	TINEA NIGRA			
**Genitourinary Conditions (Presumed To Be Infective)**							

GONOCOCCAL INFECTION OF LOWER GENITOURINARY TRACT WITHOUT PERIURETHRAL OR ACCESSORY GLAND ABSCESS	URINARY TRACT INFECTION	URINARY TRACT INFECTION, SITE NOT SPECIFIED	UTI	UNSPECIFIED SEXUALLY TRANSMITTED DISEASE	VAGINAL DISCHARGE	BALANITIS	SEXUALLY TRANSMITTED DISEASE
MALE GENITAL LESION	UTI (URINARY TRACT INFECTION)	BV (BACTERIAL VAGINOSIS)	CYSTITIS	BACTERIAL VAGINOSIS	CHRONIC PROSTATITIS	OTHER VENEREAL DISEASE	FEMALE GENITAL TRACT INFECTION
EPIDIDYMITIS	PYELONEPHRITIS	URETHRITIS	ORCHITIS	VAGINAL TRICHOMONIASIS	SYPHILIS	PELVIC INFLAMMATORY DISEASE	BALANOPOSTHITIS
NONGONOCOCCAL URETHRITIS							
**Genitourinary Conditions (Presumed To Be Non-Infective)**							
CANDIDIASIS	CANDIDIASIS, UNSPECIFIED	HAEMATURIA	UNSPECIFIED HAEMATURIA	DISORDER OF KIDNEY AND URETER, UNSPECIFIED	UNSPECIFIED CONDITION ASSOCIATED WITH FEMALE GENITAL ORGANS AND MENSTRUAL CYCLE	URINARY INCONTINENCE	UNSPECIFIED URINARY INCONTINENCE
URINARY CALCULUS, UNSPECIFIED	SEXUAL DYSFUNCTION	OTHER MALE GENITAL DISORDERS	OTHER GYNAECOLOGICAL CONDITIONS	DYSMENORRHOEA	MENORRHAGIA	CALCULUS, URINARY TRACT	OTHER URINARY DISORDERS

ABNORMAL UTERINE AND VAGINAL BLEEDING, UNSPECIFIED	CALCULUS, URINARY	DISORDER OF KIDNEY AND URETER	GENITAL HERPES (RECURRENT)	UNDESCENDED TESTICLE, UNSPECIFIED LATERALITY, UNSPECIFIED SITE	UNSPECIFIED SEXUAL DYSFUNCTION, NOT CAUSED BY ORGANIC DISORDER OR DISEASE	DISORDER OF MENSTRUAL BLEEDING	BPH ASSOCIATED WITH NOCTURIA
BPH (BENIGN PROSTATIC HYPERPLASIA)	PHIMOSIS OF PENIS	CONGENITAL ANOMALY OF URINARY SYSTEM	CONGENITAL ANOMALY OF FEMALE GENITAL SYSTEM	UTERINE FIBROID	PCOS (POLYCYSTIC OVARIAN SYNDROME)	CALCULUS OF URETER	CANDIDIASIS OF VAGINA
CANDIDIASIS OF VULVA AND VAGINA	VULVOVAGINAL CANDIDIASIS	PROLAPSE OF FEMALE PELVIC ORGANS	RENAL COLIC	URINARY DISORDER	URINE ABNORMALITY	BENIGN ESSENTIAL MICROSCOPIC HAEMATURIA	UROLITHIASIS
ALBUMINURIA	BLADDER DISORDER	CANDIDIASIS OF VULVA	POST-MENOPAUSAL ATROPHIC VAGINITIS	ATROPHIC VAGINITIS	DISORDER OF MALE GENITAL ORGAN	DISORDER OF MALE GENITAL ORGANS, UNSPECIFIED	DISORDER OF FEMALE GENITAL ORGANS
FEMALE GENITAL DISORDER	RENAL STONE	AKI (ACUTE KIDNEY INJURY)	CONGENITAL MALFORMATION OF URINARY SYSTEM, UNSPECIFIED	CALCULUS OF KIDNEY	MENSTRUAL DISORDER	URGE INCONTINENCE	URINARY RETENTION
CANDIDIASIS OF UROGENITAL SITE	POST-MENOPAUSAL BLEEDING	STRESS INCONTINENC E	GENITAL HERPES	RETENTION OF URINE	URINARY CALCULUS	IRREGULAR MENSTRUAL BLEEDING	ABNORMAL UTERINE BLEEDING

POSTCOITAL BLEEDING	NEUROGENIC BLADDER	NONINFLAMMATORY DISORDER OF VAGINA	STAGHORN CALCULUS	NONINFLAMMATORY DISORDER OF VULVA AND PERINEUM	RECURRENT GENITAL HERPES	CALCULUS IN BLADDER	
**Gastrointestinal Conditions (Presumed To Be Infective)**							
ANORECTAL ABSCESS	OTHER GASTROENTERITIS AND COLITIS OF UNSPECIFIED ORIGIN	PEPTIC ULCER, UNSPECIFIED AS ACUTE OR CHRONIC, WITHOUT HAEMORRHAGE OR PERFORATION	PEPTIC ULCER DISEASE	ACUTE APPENDICITIS UNSPECIFIED	PERINEAL ABSCESS	PERIANAL ABSCESS	HELICOBACTER PYLORI INFECTION
GASTRIC ULCER	DUODENAL ULCER	ANORECTAL FISTULA	FOREIGN BODY IN ORAL CAVITY	ANGULAR CHEILITIS	GLOSSITIS	FOREIGN BODY IN ALIMENTARY TRACT, PART UNSPECIFIED	FOREIGN BODY IN ALIMENTARY TRACT
**Gastrointestinal Conditions (Presumed To Be Non-Infective)**							
GORD (GASTRO OESOPHAGEAL REFLUX DISEASE)	GASTROESOPHAGEAL REFLUX DISEASE	GASTROENTERITIS, ACUTE	ANAL FISSURE, UNSPECIFIED	ANAL FISTULA	DYSPHAGIA	FUNCTIONAL DYSPEPSIA	GASTRODUODENITIS, UNSPECIFIED
GASTRO-OESOPHAGEAL REFLUX DISEASE WITHOUT OESOPHAGITIS	HAEMORRHOIDS	IRRITABLE BOWEL SYNDROME WITHOUT DIARRHOEA	NONINFECTIOUS GASTROENTERITIS AND COLITIS	UNSPECIFIED ABDOMINAL HERNIA WITHOUT OBSTRUCTION OR GANGRENE	UNSPECIFIED HAEMORRHOIDS WITHOUT COMPLICATION ¿¿	INCONTINENCE/ENURESIS	CONSTIPATION

GERD	PILES	GASTRITIS	OTHER GIT CONDITIONS	DYSPEPSIA	DISEASE OF INTESTINE, UNSPECIFIED	OTHER AND UNSPECIFIED ABDOMINAL PAIN	ABDOMINAL PAIN
DYSPEPSIA AND DISORDER OF FUNCTION OF STOMACH	IBS	VOMITING OF PREGNANCY, UNSPECIFIED	DISORDER OF INTESTINE	ANAL FISSURE	GASTRODUODE NITIS	ABDOMINAL HERNIA	IRRITABLE BOWEL SYNDROME
GASTROENTERITI S	PILES (HAEMORRHOID S)	DIARRHOEA	INTESTINAL DISORDER	GERD (GASTROESOPH AGEAL REFLUX DISEASE)	PERIANAL FISTULA	INTESTINAL BLEEDING	INGUINAL HERNIA
ENTERITIS,GE	ENTERITIS	CANDIDIASIS OF MOUTH	VIRAL GASTRITIS	DIVERTICULAR DISEASE	CHOLECYSTITIS	RECURRENT ORAL ULCERS	PRURITUS ANI
DIVERTICULITIS OF INTESTINE WITHOUT PERFORATION OR ABSCESS WITH BLEEDING	INFLAMMATOR Y BOWEL DISEASE	ILEOSTOMY PRESENT	ULCERATIVE COLITIS	PARASITIC INTESTINAL DISEASE	DIVERTICULOSIS OF COLON	IRRITABLE BOWEL SYNDROME WITH DIARRHOEA	CROHN'S DISEASE
LEUKOPLAKIA OF ORAL MUCOSA	HAEMATEMESIS	STATUS POST ILEOSTOMY					
**Infectious Disease Conditions (Presumed To Be Infective)**							
INFECTIOUS DISEASE	OTHER AND UNSPECIFIED INFECTIOUS DISEASES	OTHER INFECTIONS (NON-NOTIFIABLE)					
**Infectious Disease Conditions (Presumed To Be Non-Infective)**							

DENGUE FEVER [CLASSICAL DENGUE]	ENTEROVIRAL VESICULAR STOMATITIS WITH EXANTHEM	VARICELLA WITHOUT COMPLICATIO N	ZOSTER WITHOUT COMPLICATION	HERPES ZOSTER	DENGUE	CHICKENPOX	HERPES ZOSTER WITHOUT COMPLICATION
UNSPECIFIED ARTHROPOD-BORNE VIRAL FEVER	VARICELLA UNCOMPLICATE D	VIRAL ILLNESS	FEVER	HAND, FOOT AND MOUTH DISEASE (HFMD)	PARASITE INFECTION	VIRAL HEPATITIS	HERPES SIMPLEX INFECTION
HERPES STOMATITIS	HERPES ZOSTER WITH COMPLICATION	VIRAL INFECTION	ASYMPTOMATIC HIV INFECTION	VIRAL EXANTHEM	MERS-COV INFECTION	LEUKOCYTOSIS (LEUCOCYTOSIS)	
**Dental Conditions**							
NECROSIS OF PULP	REVERSIBLE PULPITIS	IRREVERSIBLE PULPITIS	PERIODONTITIS	DENTAL CARIES	GINGIVITIS	DEFECTIVE DENTAL RESTORATION	RETAINED DENTAL ROOT
COMBINED PERIODONTAL AND ENDODONTIC LESION	CARIES	TOOTH ABRASION	ARRESTED DENTAL CARIES	FRACTURE OF CROWN, ENAMEL, AND DENTIN OF TOOTH WITHOUT PULP EXPOSURE	DENTINE HYPERSENSITIVI TY	FRACTURE OF CROWN, ENAMEL, AND DENTIN OF TOOTH WITH PULP EXPOSURE	ABRASION OF TEETH
FRACTURE OF DENTAL RESTORATION	GINGIVAL HYPERPLASIA	FRACTURE OF TOOTH	TEETH PROBLEM	GUM DISEASE	DISORDER OF TEETH AND SUPPORTING STRUCTURES	DISORDER OF TEETH AND SUPPORTING STRUCTURES, UNSPECIFIED	OTHER AND UNSPECIFIED LESIONS OF ORAL MUCOSA

TEETH & SUPPORTING STRUCTURE DISEASE	DISEASE OF SALIVARY GLAND, UNSPECIFIED	DISORDER OF ORAL SOFT TISSUE	DISORDER OF SALIVARY GLAND	ORAL SOFT TISSUE DISEASE	ABSCESS OF BUCCAL SPACE OF MOUTH	IMPACTED THIRD MOLAR TOOTH	CRACKED TOOTH
IMPACTED TEETH WITH ABNORMAL POSITION	ORAL INFECTION	HORIZONTAL FRACTURE OF TOOTH	VERTICAL FRACTURE OF ROOT OF TOOTH	PERI-IMPLANTITIS, DENTAL	FASCIAL SPACE INFECTION OF MOUTH	INFECTION OF BUCCAL SPACE	FRACTURE OF ROOT OF TOOTH
PERIODONTAL ABSCESS	PERICORONITIS	SYMPTOMATIC PERIAPICAL PERIODONTITI S	ALVEOLAR OSTEITIS	SYMPTOMATIC IRREVERSIBLE PULPITIS	CHRONIC APICAL ABSCESS	PULPAL NECROSIS	APICAL ABSCESS
Dental	DENTAL ABSCESS	IMPACTED TOOTH					
**ENT Conditions (Presumed To Be Infective)**							
ACUTE TONSILLITIS	DISORDER OF EAR	DISORDER OF EAR, UNSPECIFIED	OTITIS EXTERNA, UNSPECIFIED	OTITIS MEDIA, UNSPECIFIED	OTITIS EXTERNA	OTITIS MEDIA	ACUTE SINUSITIS
CHRONIC SINUSITIS	DISORDER OF NOSE	ACUTE INFECTIVE OTITIS EXTERNA	INFECTIVE OTITIS EXTERNA	EXTERNAL OTITIS OF LEFT EAR	SINUS DISORDER	SINUSITIS	CERVICAL LYMPHADENOP ATHY
OTOMYCOSIS	ACUTE OTITIS MEDIA	ACUTE OTITIS EXTERNA	ACUTE MAXILLARY SINUSITIS	PHARYNGITIS	CHRONIC SEROUS OTITIS MEDIA	CHRONIC INFECTIVE OTITIS EXTERNA	CHOLESTEATOM A
CHRONIC NONSUPPURATI VE OTITIS MEDIA	PREAURICULAR SINUS AND CYST	PERITONSILLA R ABSCESS	FOREIGN BODY IN EAR	FOREIGN BODY IN NOSTRIL	FOREIGN BODY IN NOSE	FOREIGN BODY OF THROAT	FOREIGN BODY IN EAR LOBE
FOREIGN BODY IN THROAT							

**ENT Conditions (Presumed To Be Non-Infective)**							
ALLERGIC RHINITIS, UNSPECIFIED	EPISTAXIS	ALLERGIC RHINITIS	CHRONIC MUCOID OTITIS MEDIA	CHRONIC SECRETORY OTITIS MEDIA	IMPACTED CERUMEN	EAR WAX	OTHER EAR CONDITIONS
FB EAR	HEARING LOSS, UNSPECIFIED	HEARING LOSS	MUMPS WITHOUT COMPLICATION	PROBLEMS WITH HEARING	MUMPS	PERFORATION OF TYMPANIC MEMBRANE	THROAT DISORDER
DISORDER OF VOICE AND RESONANCE	EUSTACHIAN TUBE DISORDER	POST-NASAL DRIP	POLYP OF NASAL CAVITY	RUPTURE OF TYMPANIC MEMBRANE	DYSPHONIA	HYPERTROPHY OF NASAL TURBINATES	
**Eye Conditions (Presumed To Be Infective)**							
FB EYE	CONJUNCTIVITIS	CHALAZION	CONJUNCTIVITIS, UNSPECIFIED	EYELID DISORDER	DISORDER OF EYELID, UNSPECIFIED	FOREIGN BODY ON EXTERNAL EYE, PART UNSPECIFIED	FOREIGN BODY IN EXTERNAL EYE
BLEPHARITIS OF EYELID OF LEFT EYE	EXTERNAL HORDEOLUM	INFECTED EYE LID	HORDEOLUM	BLEPHARITIS	STYE EXTERNAL	STYE	PERIORBITAL CELLULITIS
ACUTE CONJUNCTIVITIS	CORNEAL ULCER	FOREIGN BODY IN EYE	CORNEAL ABRASION	KERATITIS	CORNEAL OPACITY	HERPES ZOSTER BLEPHARITIS	DACRYOCYSTITI S
HERPES ZOSTER KERATOCONJUN CTIVITIS	FOREIGN BODY OF EYELID						
**Eye Conditions (Presumed To Be Non-Infective)**							
DISORDER OF EYE	DISORDER OF EYE AND ADNEXA, UNSPECIFIED	DISORDER OF REFRACTION, UNSPECIFIED	GLAUCOMA, UNSPECIFIED	REFRACTIVE VISION	CATARACTS	OTHER EYE CONDITIONS	CATARACT, UNSPECIFIED

DISORDER OF EYELID	CONGENITAL ANOMALY OF EYE	EYE DISORDER	CATARACT	DRY EYES	GLAUCOMA	EYE DISCOMFORT	DISORDER OF REFRACTION AND ACCOMMODATION
BLINDNESS OF ONE EYE	CONJUNCTIVAL HAEMORRHAGE	H/O SUBCONJUNCTIVAL HAEMORRHAGE	VITREOUS HAEMORRHAGE OF LEFT EYE	CONGENITAL MALFORMATION OF EYE, UNSPECIFIED	EYE STRAIN	RED EYE	TRAUMA TO EYE
MYOPIA							
**Undefined**							
IMPAIRED GLUCOSE REGULATION	IMPAIRED GLUCOSE REGULATION WITHOUT COMPLICATION	IMPAIRED GLUCOSE TOLERANCE	PERSONAL HISTORY OF LONG-TERM (CURRENT) USE OF OTHER MEDICAMENTS, INSULIN	TYPE 1 DIABETES MELLITUS WITHOUT COMPLICATION	TYPE 2 DIABETES MELLITUS	TYPE 2 DIABETES MELLITUS WITHOUT COMPLICATION	UNSPECIFIED DIABETES MELLITUS WITH BACKGROUND RETINOPATHY
UNSPECIFIED DIABETES MELLITUS WITH HYPOGLYCAEMIA	IMPAIRED FASTING GLUCOSE(IFG)	DM RETINOPATHY	IMPAIRED GLUCOSE TOLERANCE(IGT)	DM NEUROPATHY	DM NEPHROPATHY - ESRF ON DIALYSIS	DM TYPE I ON MEDICATION	DM TYPE II ON MEDICATION
DM NEPHROPATHY - OVERT	DM NEPHROPATHY - INCIPIENT	DM TYPE II (DIET ONLY)	CURRENT USE OF INSULIN	DIABETES MELLITUS WITH INCIPIENT DIABETIC NEPHROPATHY	DIABETES MELLITUS WITH RETINOPATHY	IMPAIRED FASTING GLUCOSE	HYPOGLYCAEMIA

TYPE 2 DIABETES MELLITUS WITH HYPEROSMOLARITY WITH COMA	DIABETES MELLITUS, TYPE II	DIABETES MELLITUS	DIABETIC KIDNEY DISEASE	IFG (IMPAIRED FASTING GLUCOSE)	TYPE 1 DIABETES MELLITUS	TYPE 2 DIABETES MELLITUS WITH HYPEROSMOLAR COMA	HYPOGLYCAEMIA ASSOCIATED WITH DIABETES
DIABETIC RETINOPATHY	DM (DIABETES MELLITUS)	DIABETIC NEUROPATHY	LONG TERM CURRENT USE OF INSULIN	T2DM (TYPE 2 DIABETES MELLITUS)	IGT (IMPAIRED GLUCOSE TOLERANCE)	TYPE 2 DIABETES MELLITUS WITH COMPLICATIONS	COMPLICATION OF PROCEDURE
BIPOLAR DISORDER	HEART FAILURE	INFLAMMATORY ARTHROPATHY	CEREBROVASCULAR ACCIDENT (CVA)	END STAGE CHRONIC KIDNEY DISEASE	CONGENITAL ABNORMALITY	PSORIATIC ARTHROPATHY	DISORDER OF THYROID
CKD (CHRONIC KIDNEY DISEASE)	LYMPHADENOPATHY	FOOD POISONING	THALAMIC HAEMORRHAGE	BURSITIS	ACQUIRED ABSENCE OF FOOT	PERIPHERAL VENOUS INSUFFICIENCY	ARTHRALGIA
VENOUS EMBOLISM AND THROMBOSIS	VOMITING AS REASON FOR CARE IN PREGNANCY	COMPLICATION RELATED TO PREGNANCY	UNWANTED PREGNANCY	COBALAMIN DEFICIENCY	FOLIC ACID DEFICIENCY	LATERAL EPICONDYLITIS (TENNIS ELBOW)	SYNOVITIS AND TENOSYNOVITIS
TENDINITIS	TRANSIENT ISCHEMIC ATTACK	FATTY LIVER	NECK ACHE	BENIGN NEOPLASM	WELL ADULT EXAM	FRACTURE NECK OF FEMUR	ABNORMAL BONE DENSITY SCREENING
IDIOPATHIC PERIPHERAL NEUROPATHY	BENIGN NEOPLASTIC DISEASE	PARKINSON DISEASE	DIETARY COUNSELLING AND SURVEILLANCE	NON-COMPLIANCE WITH TREATMENT	COGNITIVE DYSFUNCTION	TRIGGER FINGER	MOOD DISORDER
HYPERTHYROIDISM	THALASSAEMIA	ALLERGIC DRUG REACTION	ELECTIVE SURGICAL PROCEDURE	DISORDER OF BRAIN	MAJOR DEPRESSION	MEMORY IMPAIRMENT	STAGE 4 CHRONIC KIDNEY DISEASE

PERSISTENT DELUSIONAL DISORDER	SMOKER	ISCHEMIC CEREBROVASCULAR ACCIDENT (CVA)	CONCUSSION	DEEP VEIN THROMBOSIS	ANXIETY STATE	STRAIN OF KNEE	DYSFUNCTION, PSYCHOSEXUAL
PERIPHERAL NEUROPATHY	LIPID DISORDER	FOLLOW UP	LIMB ISCHAEMIA	NONRHEUMATIC AORTIC VALVE STENOSIS	ALCOHOL ABUSE	HYPOKALAEMIA	POSTURAL HYPOTENSION
ENCOUNTER FOR EDUCATION	STATUS POST BELOW-KNEE AMPUTATION	MEDICAL CARE COMPLICATION	DE QUERVAIN'S TENOSYNOVITIS	FROZEN SHOULDER	DISORDER OF GALLBLADDER	CONGENITAL ANOMALY OF MUSCULOSKELETAL SYSTEM	BACK PAIN
DYSLIPIDAEMIA	ITCH	LOW BACK PAIN	TIA (TRANSIENT ISCHAEMIC ATTACK)	MAMMOGRAM ABNORMAL	DISEASE OF CIRCULATORY SYSTEM	OSA (OBSTRUCTIVE SLEEP APNOEA)	PALPITATIONS
METASTATIC MALIGNANT NEOPLASM	THYROID NODULE	DEPRESSIVE ILLNESS	STROKE, HAEMORRHAGIC	PRENATAL CONSULT	CHRONIC GLOMERULONEPHRITIS	SPRAIN AND STRAIN	OPTIONAL SURGERY
ENCOUNTER FOR POSTNATAL VISIT	FEMALE INFERTILITY	COMPLICATION OF THE PUERPERIUM, POSTPARTUM	ENGORGEMENT OF BREASTS ASSOCIATED WITH CHILDBIRTH, DELIVERED	SUBFERTILITY OF COUPLE	MOOD AND AFFECT DISTURBANCE	INJURED IN ROAD TRAFFIC ACCIDENT	GAD (GENERALISED ANXIETY DISORDER)

NORMAL PSYCHIATRIC ASSESSMENT	WELL CHILD CHECK	ENCOUNTER FOR EXAMINATION FOR ADOLESCENT DEVELOPMEN T STATE	ORTHOSTATIC HYPOTENSION	ASYMPTOMATI C HUMAN IMMUNODEFIC IENCY VIRUS [HIV] INFECTION STATUS	VENOUS INSUFFICIENCY (CHRONIC) (PERIPHERAL)	SCREENING FOR CONDITION	DISORDER OF ENDOCRINE SYSTEM
NUTRITIONAL DEFICIENCY DISORDER	ERRONEOUS ENCOUNTER--DISREGARD	ANAEMIA	CHRONIC ISCHAEMIC HEART DISEASE	ALLERGY	DISORDER OF BREAST	DISORDER OF CELLULAR COMPONENT OF BLOOD	CHEST PAIN
GOUT	GOUT, UNSPECIFIED, SITE UNSPECIFIED	ACQUIRED ABSENCE OF FOOT AND ANKLE	ACQUIRED ABSENCE OF LEG ABOVE KNEE	ACQUIRED ABSENCE OF LEG AT OR BELOW KNEE	ADMINISTRATIV E ENCOUNTER	ALLERGY, UNSPECIFIED	ANAEMIA, UNSPECIFIED
ARTHROPATHY	MYALGIA, SITE UNSPECIFIED	ARTHROSIS, UNSPECIFIED, SITE UNSPECIFIED	ATHEROSCLEROSIS OF ARTERIES OF EXTREMITIES	ATRIAL FIBRILLATION	ATRIAL FIBRILLATION AND FLUTTER	BACK ACHE	BELL'S PALSY
BENIGN NEOPLASM OF UNSPECIFIED SITE	BREAST LUMP	CARDIAC ARRHYTHMIA, UNSPECIFIED	CARPAL TUNNEL SYNDROME	OTHER CVS CONDITIONS	CHEST PAIN, UNSPECIFIED	CHRONIC ISCHAEMIC HEART DISEASE, UNSPECIFIED	CHRONIC NEPHRITIC SYNDROME, UNSPECIFIED
CHRONIC LIVER DISEASE	CONDITION ORIGINATING IN THE PERINATAL PERIOD, UNSPECIFIED	CONGENITAL MALFORMATI ON OF HEART, UNSPECIFIED	CONGENITAL MALFORMATION OF MUSCULOSKELETA L SYSTEM, UNSPECIFIED	CONGESTIVE HEART FAILURE	ARTHRALGIA & MYALGIA	CONTUSION OF UNSPECIFIED BODY REGION	COUNSELLING, UNSPECIFIED

DELAYED MILESTONE	DEPRESSIVE EPISODE, UNSPECIFIED, NOT SPECIFIED AS ARISING IN THE POSTNATAL PERIOD	DISEASE OF BLOOD AND BLOOD-FORMING ORGANS, UNSPECIFIED	DISEASE OF GALLBLADDER, UNSPECIFIED	DISLOCATION, SPRAIN AND STRAIN OF UNSPECIFIED BODY REGION	DISORDER OF BRAIN, UNSPECIFIED	DISORDER OF HEART	DISORDERS OF INITIATING AND MAINTAINING SLEEP [INSOMNIAS]
DIZZINESS AND GIDDINESS	DOWN'S SYNDROME, UNSPECIFIED	ELEVATED BLOOD PRESSURE READING WITHOUT DIAGNOSIS OF HYPERTENSIO N	ELEVATED BLOOD-PRESSURE READING, WITHOUT DIAGNOSIS OF HYPERTENSION	EMBOLISM AND THROMBOSIS OF UNSPECIFIED VEIN	ENCOUNTER FOR FOLLOW-UP IN OUTPATIENT CLINIC	ENDOCRINE DISORDER, UNSPECIFIED	EPILEPSY, UNSPECIFIED, WITHOUT MENTION OF INTRACTABLE EPILEPSY
ESSENTIAL (PRIMARY) HYPERTENSION	FATTY (CHANGE OF) LIVER, NOT ELSEWHERE CLASSIFIED	FEMALE INFERTILITY, UNSPECIFIED	FOLLOW-UP EXAMINATION AFTER UNSPECIFIED TREATMENT FOR OTHER CONDITIONS	FRACTURE OF UNSPECIFIED BODY REGION, CLOSED	GENERAL COUNSELLING AND ADVICE ON CONTRACEPTIO N	GENERAL MEDICAL EXAMINATION	GENERALISED ANXIETY DISORDER
GENERALISED OSTEOARTHRITIS	GYNAECOLOGIC AL EXAMINATION (GENERAL)(ROU TINE)	HEADACHE	HEART DISEASE, UNSPECIFIED	HEREDITARY AND IDIOPATHIC NEUROPATHY, UNSPECIFIED	HYPERLIPIDAEM IA	HYPERLIPIDAEMIA , UNSPECIFIED	HYPERPLASIA OF PROSTATE
HYPERTENSION	HYPOTHYROIDIS M, UNSPECIFIED	IHD (ISCHAEMIC HEART DISEASE)	ILL-DEFINED CONDITION	INAPPROPRIAT E DIET AND EATING HABITS	ISOLATED PROTEINURIA	LIVER DISEASE, UNSPECIFIED	LOSS OF CONSCIOUSNES S OF

							UNSPECIFIED DURATION
MALIGNANT NEOPLASM	MALIGNANT NEOPLASM WITHOUT SPECIFICATION OF SITE	MENOPAUSAL AND PERIMENOPAUSAL DISORDER, UNSPECIFIED	MENOPAUSAL AND POSTMENOPAUSAL DISORDER	MENTAL AND BEHAVIOURAL DISORDERS DUE TO USE OF ALCOHOL, ACUTE INTOXICATION	MIGRAINE, UNSPECIFIED	MILD COGNITIVE DISORDER	NEUROTIC DISORDER, UNSPECIFIED
NUTRITIONAL DEFICIENCY, UNSPECIFIED	OBESITY	OBESITY DUE TO EXCESS CALORIES	OBESITY, UNSPECIFIED	OSTEOARTHRITIS	OTHER AMNESIA	OTHER AND UNSPECIFIED DISORDERS OF BREAST ASSOCIATED WITH CHILDBIRTH, WITHOUT MENTION OF ATTACHMENT DIFFICULTY	OTHER AND UNSPECIFIED DISORDERS OF CIRCULATORY SYSTEM
OTHER EXAMINATIONS FOR ADMINISTRATIVE PURPOSES	OTHER GENERAL SYMPTOMS AND SIGNS	OTHER SPECIFIED COUNSELLING	OTHER SPECIFIED DISORDERS OF BREAST	OVERWEIGHT	PARKINSON'S DISEASE	PERIPHERAL VASCULAR DISEASE	PERIPHERAL VASCULAR DISEASE, UNSPECIFIED

PERSONAL HISTORY OF NONCOMPLIANCE WITH MEDICAL TREATMENT AND REGIMEN	PERSONAL HISTORY OF OTHER MENTAL AND BEHAVIOURAL DISORDERS	PLANTAR FASCIAL FIBROMATOSIS	PLANTAR FASCIITIS	POLYARTHROSIS, UNSPECIFIED	POLYNEUROPATHY, UNSPECIFIED	PROBLEMS RELATED TO UNWANTED PREGNANCY	PROCEDURE FOR PURPOSES OTHER THAN REMEDYING HEALTH STATE, UNSPECIFIED
PROPHYLACTIC MEASURE, UNSPECIFIED	PROTEINURIA	RHEUMATOID ARTHRITIS, UNSPECIFIED, SITE UNSPECIFIED	RISK FOR FALLS	ROUTINE CHILD HEALTH EXAMINATION	ROUTINE POSTPARTUM FOLLOW-UP	SCHIZOPHRENIA, UNSPECIFIED	SEVERE DEPRESSIVE EPISODE WITHOUT PSYCHOTIC SYMPTOMS, NOT SPECIFIED AS ARISING IN THE POSTNATAL PERIOD
SPECIAL SCREENING	SPECIAL SCREENING EXAMINATION, UNSPECIFIED	SPRAIN, STRAIN	STROKE	STROKE, NOT SPECIFIED AS HAEMORRHAGE OR INFARCTION	SUPERVISION OF NORMAL PREGNANCY, UNSPECIFIED	TENDENCY TO FALL, NEC	THALASSAEMIA, UNSPECIFIED
THYROTOXICOSIS, UNSPECIFIED	TOBACCO USE, CURRENT	TRANSIENT CEREBRAL ISCHAEMIC ATTACK, UNSPECIFIED	TRIGEMINAL NEURALGIA	UNSPECIFIED ADVERSE EFFECT OF DRUG OR MEDICAMENT	UNSPECIFIED DEMENTIA	UNSPECIFIED DISORDER OF BONE DENSITY AND STRUCTURE, SITE UNSPECIFIED	UNSPECIFIED DORSALGIA, SITE UNSPECIFIED

UNSPECIFIED LUMP IN BREAST	UNSPECIFIED MENTAL DISORDER DUE TO BRAIN DAMAGE AND DYSFUNCTION AND TO PHYSICAL DISEASE	UNSPECIFIED NONORGANIC PSYCHOSIS	UNSPECIFIED OSTEOPOROSIS, SITE UNSPECIFIED	UNSPECIFIED SYNOVITIS AND TENOSYNOVITIS, SITE UNSPECIFIED	CHRONIC ISCHEMIC HEART DISEASE	THYROTOXICOSIS	BACKACHE
ANXIETY	BENIGN NEOPLASMS	HEAD INJURY	CCF	SPINAL DISORDER	PSYCHOSIS	HYPERLIPIDEMIA ON MEDICATION	STROKE (NOT SPECIFIED)
THALASSEMIA	OTHER DEFORMITIES OF ANKLE AND FOOT	PVD	NUTRITIONAL DEF.	OTHER BLOOD DISORDERS	GLOMERULONEPHRITIS	OTHER MSK CONDITIONS	GIDDINESS, NOT SPECIFIED
FRACTURES	FOLLOW-UP POST SURG	OTHER PSYCHIATRIC CONDITIONS	BUNION / HALLUX VALGUS	HYPOTHYROIDISM	GRAVES' DISEASE	CHEST PAIN NOS	BIPOLAR DISORDERS
OSTEOPOROSIS - FRACTURE-VERTEBRAL	STROKE (INFARCT)	TIA	HYPERTENSION (DIET ONLY)	EPILEPSY	SPRAIN/STRAIN	WELL WOMEN CLINIC	HYPERLIPIDEMIA (DIET ONLY)
NON – DM NEPHROPATHY – INCIPIENT	OTHER SCREENING/GROWTH MONITORING. QUESTIONNAIRES	INSOMNIA	MED EXAM/INVESTIGATIONS	SCHIZOPHRENIA	OSTEOPOROSIS	ANTENATAL CARE	ACUTE ISCHEMIC HEART DISEASE
HEALTH EDUCATION	MALIGNANT NEOPLASMS	ANEMIA (EXCEPT THAL.)	CODE NOT IN DIMENSION	TRAVEL CLINIC	STROKE (HAEMORRHAGE)	DEMENTIA	DEPRESSION (OTHERS)

NON – DM NEPHROPATHY – OVERT	PARKINSONISM	MIGRAINE	PREVENTIVE MEASURES/ IMMUNISATION CHILD	HEADACHE, NOT SPECIFIED	DRP	HERNIA	FOLLOW-UP EXAM.
NEPHRITIS (EG GLOMERULONEP HRITIS)	OTHER CNS CONDITIONS	RHEUMATOID ARTHRITIS	NEPHRITIS, NEPHROPATHY, UNSPECIFIED	HYPERTENSION ON MEDICATION	BPH	CONGENITAL HEART ANOMALY	DEPRESSION (MAJOR)
COMPLICATION OF MEDICAL CARE	BASIC HEALTH SCREEN	DISORDER OF SYNOVIUM, TENDON & BURSA	OTHER ENDOCRINE DISEASES	DRUG/ALCOHO L ABUSE	NEED FOR IMMUNISATION AGAINST INFLUENZA	ENCOUNTER FOR GYNECOLOGICAL EXAMINATION	CHRONIC KIDNEY DISEASE, UNSPECIFIED
CKD STAGE 3 OR 4 (EGFR 15-59)	CKD STAGE 5 / ESRF (EGFR <15)	RENAL FAILURE, CHRONIC	CKD STAGE 4 (EGFR 15-29)	CHRONIC RENAL FAILURE	CHRONIC RENAL INSUFFICIENCY, STAGE III (MODERATE)	CKD STAGE 2 (EGFR 60-89)	OSTEOPENIA
ACHILLES TENDINITIS	CEREBRAL PALSY	ARRHYTHMIAS	THYROIDITIS	GALL BLADDER DISEASE	ADVERSE EFFECT, MEDICATION, CHEMICAL	IRON DEFICIENCY	MENOPAUSAL DISORDERS
FAMILY PLANNING	PES PLANUS	DOWN'S SYNDROME	OTHER RENAL DISORDERS	UNSPECIFIED MENTAL RETARDATION WITHOUT MENTION OF IMPAIRMENT OF BEHAVIOUR	ADJUSTMENT DISORDERS	BIPOLAR AFFECTIVE DISORDER, UNSPECIFIED	UNSPECIFIED COMPLICATION OF PROCEDURE

CONTACT WITH AND EXPOSURE TO OTHER COMMUNICABLE DISEASES	NEED FOR IMMUNISATION AGAINST UNSPECIFIED COMBINATIONS OF INFECTIOUS DISEASES	RADIAL STYLOID TENOSYNOVITIS [DE QUERVAIN]	OTHER AND UNSPECIFIED ABNORMALITIES OF GAIT AND MOBILITY	UNSPECIFIED HARMFUL USE OF NON-DEPENDENCE PRODUCING SUBSTANCE	EXAMINATION FOR ADOLESCENT DEVELOPMENT STATE	OTHER PROBLEMS RELATED TO HOUSING AND ECONOMIC CIRCUMSTANCES	OTHER SPECIFIED POSTPROCEDURAL STATES;PREVIOUSLY INITIATED ENDODONTIC THERAPY COMPLETED
OTHER SPECIFIED PROPHYLACTIC MEASURES	PERSISTENT DELUSIONAL DISORDER, UNSPECIFIED	PREGNANCY-RELATED CONDITION, UNSPECIFIED	CEREBRAL PALSY, UNSPECIFIED	COGNITIVE IMPAIRMENT	POSTOPERATIVE FOLLOW-UP	MYALGIA	DELAYED DEVELOPMENTAL MILESTONES
GENERAL COUNSELLING AND ADVICE FOR CONTRACEPTIVE MANAGEMENT	CLOSED FRACTURE	VERTIGO	FALL	ADVERSE EFFECT OF DRUG OR MEDICAMENT	ESRF (END STAGE RENAL FAILURE)	CARRIER OF VIRAL HEPATITIS B	UNSPECIFIED VIRAL HEPATITIS WITHOUT HEPATIC COMA
HEP B CARRIER FOLLOW-UP	HEPATITIS B CARRIER	HEPATITIS B INFECTION	DISAPPEARANCE AND DEATH OF FAMILY MEMBER	LACK OF PHYSICAL EXERCISE	COMPLICATION OF SURGICAL AND MEDICAL CARE, UNSPECIFIED	TINNITUS	SUBCLINICAL HYPERTHYROIDISM
CARCINOMA OF BREAST	MENOPAUSE	FIBROID	TREMOR	CALCANEAL SPUR	DRY EYE	MYELOPATHY DUE TO INTERVERTEBRAL DISC DISEASE	VITAMIN D DEFICIENCY
OA (OSTEOARTHRITIS) OF KNEE	ELECTROLYTE ABNORMALITY	SPONDYLOSIS, CERVICAL	NORMOCYTIC NORMOCHROMIC ANAEMIA	HYPERLIPAEMIA	ANXIETY DISORDER	ACQUIRED HALLUX VALGUS OF RIGHT FOOT	ADHESIVE CAPSULITIS OF RIGHT SHOULDER

MULTIPLE SCLEROSIS	LIVER CIRRHOSIS	NEOPLASM OF SUBMANDIBULAR SALIVARY GLAND	POST HERPETIC NEURALGIA	CARCINOMA OF COLON	TRAVEL ADVICE ENCOUNTER	LEFT KNEE PAIN	ACHILLES TENDINITIS OF LEFT LOWER EXTREMITY
MUSCULOSKELETAL PAIN	ADHESIVE CAPSULITIS OF LEFT SHOULDER	SUBCLINICAL HYPOTHYROIDISM	CORONARY ARTERY DISEASE	CARCINOMA OF GALLBLADDER	NONTOXIC NODULAR GOITRE	MILD DEPRESSION	SPINAL STENOSIS OF LUMBAR REGION
TRIGGER THUMB OF RIGHT HAND	ERECTILE DYSFUNCTION	SWALLOWING DIFFICULTY	ABNORMAL WEIGHT LOSS	CHANGE IN BOWEL HABITS	VITAMIN B DEFICIENCY	VISION BLURRING	LUMBAR SPONDYLOSIS
OSTEOPOROSIS WITH PATHOLOGICAL FRACTURE	THALASSAEMIA TRAIT	ARMD (AGE RELATED MACULAR DEGENERATION)	TRIGGER FINGER OF RIGHT HAND	DISORDER OF FLUID OR ELECTROLYTE	CARCINOMA OF LUNG	CARCINOMA OF NASOPHARYNX	CARCINOMA OF PROSTATE
SENSORINEURAL HEARING LOSS (SNHL)	ROTATOR CUFF SYNDROME	CARDIOMYOPATHY	KNEE PAIN	SHOULDER PAIN	SPINAL STENOSIS	PRESBYCUSIS	THROMBOCYTOPAENIA
VASCULAR DEMENTIA	SPONDYLOSIS	UMBILICAL HERNIA	COSTOCHONDRITIS	SIADH (SYNDROME OF INAPPROPRIATE ADH PRODUCTION)	LYMPHOMA	CARCINOMA OF BLADDER	ISCHAEMIC CARDIOMYOPATHY
HASHIMOTO'S THYROIDITIS	TRIGGER FINGER OF LEFT HAND	RHEUMATOID ARTHRITIS WITH NEGATIVE RHEUMATOID FACTOR	DELIRIUM	CARCINOMA OF STOMACH	BETA THALASSAEMIA MINOR	ACUTE KIDNEY FAILURE	HIP PAIN

VARICOSE VEIN OF SCROTUM	HYPERCALCAEM IA	EXERTIONAL DYSPNOEA	ABNORMAL BLOOD TEST	HYPERKALAEMI A	FLUID OVERLOAD	INFLUENZA VACCINATION ADMINISTERED AT CURRENT VISIT	ADVERSE EFFECT OF VACCINE
DISORDER OF ARTERY OR ARTERIOLE	MALIGNANT NEOPLASM OF COLON	CHRONIC URTICARIA	DEXTROCARDIA	DISEASE OF PITUITARY GLAND	PANIC DISORDER	DYSPNOEA	VERTEBROBASIL AR TIAS
TYPE 1 NEUROFIBROMA TOSIS	LESION OF ULNAR NERVE	RIGHT KNEE PAIN	LEFT SHOULDER PAIN	SICK SINUS SYNDROME	HEPATOMEGAL Y	PERSISTENT PROTEINURIA	NONTOXIC SINGLE THYROID NODULE
AORTIC STENOSIS	MAJOR DEPRESSIVE DISORDER	UTEROVAGINA L PROLAPSE	ALCOHOLIC LIVER DISEASE	ANAEMIA OF CHRONIC DISEASE	CARDIAC ARRHYTHMIA	CARPAL TUNNEL SYNDROME OF RIGHT WRIST	ABNORMAL PAPANICOLAOU SMEAR OF VAGINA
OVARIAN CYST	LIVER CANCER	OSTEOARTHRI TIS OF HAND	LIVES ALONE	HEPATOCELLUL AR CARCINOMA	ACQUIRED TRIGGER FINGER	PTERYGIUM OF EYE	BEREAVEMENT
AORTIC REGURGITATION	ESSENTIAL TREMOR	RADICULOPAT HY, LUMBAR REGION	LATERAL EPICONDYLITIS OF RIGHT ELBOW	HYPOCALCAEM IA	SCOLIOSIS	TRIGGER THUMB OF LEFT HAND	DIALYSIS PATIENT
RIGHT SHOULDER PAIN	LEFT TENNIS ELBOW	MEDIAL EPICONDYLITIS OF RIGHT ELBOW	TENSION HEADACHE	GLAUCOMA SUSPECT	SUBJECTIVE MEMORY COMPLAINTS	ROTATOR CUFF INJURY	MEDIAL EPICONDYLITIS OF LEFT ELBOW
LATERAL EPICONDYLITIS OF LEFT ELBOW	PIGMENTED SKIN LESION	BILATERAL SHOULDER PAIN	INTERSTITIAL LUNG DISEASE	MULTIPLE MYELOMA	MALIGNANT THYMOMA	HALLUX VALGUS, ACQUIRED	ATRIAL FLUTTER

FRACTURE OF SPINE	INCISIONAL HERNIA	MEDIAL EPICONDYLITIS	LOW GRADE SQUAMOUS INTRAEPITHELIAL LESION (LGSIL) ON PAPANICOLAOU SMEAR OF CERVIX	CYST, KIDNEY, ACQUIRED	CHRONIC HEPATITIS C VIRUS INFECTION	PTOSIS	ABNORMAL FINDINGS ON DIAGNOSTIC IMAGING OF BREAST
FOOD ALLERGY	NEPHROTIC SYNDROME	NEED FOR IMMUNISATIO N AGAINST COMBINATION OF INFECTIOUS DISEASES	PERINATAL DISORDER	CONNECTIVE TISSUE DISORDER	EDUCATIONAL PROBLEM	ABNORMAL SCREENING TEST FOR CERVICAL CANCER	CARPAL TUNNEL SYNDROME OF LEFT WRIST
ACUTE STRESS REACTION	AUTOIMMUNE DISEASE	DEPRESSIVE EPISODE ARISING IN POSTNATAL PERIOD	DISORDER OF TENDON	DOWN SYNDROME	PSYCHOTIC DISORDER	THROMBOCYTOSI S	PERSONALITY DISORDER
ENDOMETRIOSIS	ACHILLES TENDINITIS OF RIGHT LOWER EXTREMITY	AMENORRHOE A	ADJUSTMENT DISORDER	SLEEP APNOEA	ALOPECIA, SCARRING	GENERALISED SKIN ERUPTION DUE TO DRUGS AND MEDICAMENTS	NON-SMALL CELL LUNG CANCER
CEREBRAL ANEURYSM	SEIZURE	ACQUIRED HALLUX VALGUS OF LEFT FOOT	SYSTEMIC LUPUS ERYTHEMATOSUS	BEHAVIOURAL CHANGE	COGNITIVE DECLINE	RIGHT HIP PAIN	ALPHA THALASSAEMIA MINOR

FRACTURE WITH NONUNION	AMI (ACUTE MYOCARDIAL INFARCTION)	DEVIATED NASAL SEPTUM	FRACTURE OF LEFT HIP	UPPER RESPIRATORY TRACT HYPERSENSITIVITY REACTION	LOSS OF CONSCIOUSNESS	DISORDER OF REFRACTION	MITRAL VALVE DISEASE
PHOBIA	JAUNDICE	BRADYCARDIA	TRIGGER FINGER OF BOTH HANDS	TORTICOLLIS	CARCINOMA OF THYROID GLAND	KIDNEY TRANSPLANTED	IRREGULAR MENSTRUAL CYCLE
INTELLECTUAL DISABILITY	ABNORMAL RESULTS OF CARDIOVASCULAR FUNCTION STUDIES	CHRONIC PAIN	MYASTHENIA GRAVIS	PAIN IN BOTH KNEES	VITILIGO	CERVICAL POLYP	CERVICAL CANCER SCREENING
PAP SMEAR ABNORMALITY OF CERVIX/HUMAN PAPILLOMAVIRUS (HPV) POSITIVE	DEGENERATION OF INTERVERTEBRAL DISC	DYSPAREUNIA	BULIMIA NERVOSA	MULTIPLE CLOSED JOINT DISLOCATIONS	FRACTURE OF HIP	RAYNAUD'S PHENOMENON	MELAENA
AORTIC ANEURYSM WITHOUT RUPTURE	DIABETIC MACULOPATHY	ALTERED GROWTH AND DEVELOPMENT	SUBLUXATION	CARDIAC MURMUR	SPONDYLOSIS OF LUMBAR SPINE	ANKYLOSING SPONDYLITIS	SIMPLE GOITRE
RIGHT TENNIS ELBOW	CONGENITAL HEART DISEASE	RETINAL TEAR	POLYP OF COLON	COMPLETE UTERINE PROLAPSE	MENIERE'S DISEASE	ANTIPHOSPHOLIPID SYNDROME	DIABETES INSIPIDUS

SPINE DISORDER	ANGINA PECTORIS	SINUS TACHYCARDIA	ALOPECIA AREATA	ACCESSORY BREAST	GESTATIONAL DIABETES MELLITUS (GDM)	CONGENITAL ANOMALY OF MALE GENITAL SYSTEM	RESPIRATORY FAILURE
HEPATITIS C VIRUS CARRIER STATE	POLYCYSTIC OVARY SYNDROME	INFANTILE CEREBRAL PALSY	FAMILIAL HYPERCHOLESTER OLAEMIA	POLYCYSTIC KIDNEY DISEASE	MITRAL REGURGITATIO N	DERMATOMYOSIT IS	LID RETRACTION
OPEN FRACTURE	FRACTURE OF RIGHT HIP	VENTRICULAR BIGEMINY	SUICIDAL THOUGHTS	AGE-RELATED MACULAR DEGENERATIO N	BASAL CELL CARCINOMA (BCC)	DEHYDRATION	DRUG ABUSE
CARCINOMA OF UTERUS	MITRAL VALVE PROLAPSE	SJOGREN'S SYNDROME	OVERFLOW INCONTINENCE	ANAL POLYP	HAMMER TOE	DYSARTHRIA	ADENOMA
MIXED HYPERLIPIDAEMI A	XANTHELASMA OF EYELID	MULTINODUL AR THYROID	GOLFERS ELBOW OF RIGHT UPPER EXTREMITY	CAPILLARY HAEMANGIOM A	MILD DEPRESSIVE EPISODE	MODERATE DEPRESSIVE EPISODE	STRABISMUS
HIP FRACTURE, OSTEOPOROTIC	VERTEBRAL FRACTURE, OSTEOPOROTIC	PRIMARY ALDOSTERONI SM	PAP SMEAR OF CERVIX WITH ASCUS, CANNOT EXCLUDE HGSIL	BRAIN TUMOUR	HEPATIC CIRRHOSIS DUE TO CHRONIC HEPATITIS C INFECTION	NERVE COMPRESSION SYNDROME	ARRHYTHMIA
PROPHYLACTIC MEASURE	MALIGNANT NEOPLASTIC DISEASE	ALCOHOLIC FATTY LIVER	NONALCOHOLIC STEATOHEPATITIS (NASH)	CONGENITAL MALFORMATIO N OF EAR	DIFFICULTY USING HEARING AID	UNDERWEIGHT	CHRONIC PANCREATITIS
MALLET FINGER OF RIGHT HAND	TYPE 2 DIABETES MELLITUS WITH DIABETIC NEPHROPATHY	OROFACIAL DYSKINESIA	PTSD (POST-TRAUMATIC STRESS DISORDER)	SPONDYLOART HRITIS	BILATERAL CARPAL TUNNEL SYNDROME	PROLIFERATIVE DIABETIC RETINOPATHY	INJURY OF LEFT ROTATOR CUFF

NEUROTIC DISORDER	HIRSUTISM	CARDIAC SEPTAL DEFECT	HYPEREMESIS GRAVIDARUM	DISORDER OF MINERAL METABOLISM	ABNORMAL CERVICAL PAPANICOLAOU SMEAR	BLEEDING IN EARLY PREGNANCY	ODYNOPHAGIA
CONGENITAL HYPOTHYROIDIS M	ANOREXIA NERVOSA	FINANCIAL PROBLEMS	ALTERED MENTAL STATE	RHEUMATIC HEART DISEASE	LEFT HIP PAIN	RENAL CELL CARCINOMA	INJURY OF RIGHT ROTATOR CUFF
CONDUCTIVE HEARING LOSS	IDIOPATHIC THROMBOCYTO PAENIC PURPURA (ITP)	SPLENOMEGAL Y	INTERVERTEBRAL DISC DISORDER	DIABETES MELLITUS WITH OPHTHALMIC MANIFESTATIO N	CYSTOCELE WITHOUT UTERINE PROLAPSE	DRY MOUTH	PELVIC MASS
HYPERTRICHOSIS	DUPLEX KIDNEY	POLYP OF GALLBLADDER	REPETITIVE STRESS INJURY	TELOGEN EFFLUVIUM	COLLAPSED VERTEBRA DUE TO OSTEOPOROSIS	LEUKOPENIA	ACS (ACUTE CORONARY SYNDROME)
DIABETIC NEPHROPATHY	BEHAVIOURAL AND EMOTIONAL DISORDERS WITH ONSET USUALLY OCCURRING IN CHILDHOOD AND ADOLESCENCE	MALLET FINGER OF LEFT HAND	CHONDROMALACI A OF RIGHT PATELLA	GOLFER'S ELBOW	CLOSED DISLOCATION		
